# Love sickness: gender differences in the neurobiology and psychopathology of romantic suffering

**DOI:** 10.1192/j.eurpsy.2026.11307

**Published:** 2026-07-31

**Authors:** A. C. Matias, T. Vieira, J. Hortet, S. D. Duarte, F. M. Santos, P. F. Coelho, R. M. Lopes, A. F. Silva, L. Ferreira, L. Delgado

**Affiliations:** Unidade Local de Saúde do Médio Tejo, Tomar, Portugal

## Abstract

**Introduction:**

Romantic attachment is a fundamental human drive supported by neural systems involved in reward, motivation, and social bonding. When disrupted by loss or rejection, these same circuits contribute to what is often described as ”love sickness”—a state characterized by emotional distress, obsessive thinking, and physiological arousal. Neuroimaging research indicates that romantic suffering engages brain regions implicated in both physical pain and affective disorders, including the ventrolateral prefrontal cortex (vlPFC), anterior cingulate cortex (ACC), and anterior insula (AI). Beyond neurobiology, romantic loss has been linked to psychopathological symptoms such as depression, anxiety, and exacerbating borderline personality symptoms.

**Objectives:**

The objective of this review is to examine the neurobiological correlates of romantic suffering, explore gender differences in neural and psychological responses to romantic rejection or loss and discuss the psychopathological implications of romantic distress, particularly its links to depression, anxiety, and emotional dysregulation.

**Methods:**

A non-systematic review of the literature, using combinations of keywords such as “romantic rejection”, “love sickness”, “neurobiology”, “gender differences”, “social pain” and “psychopathology” in database PubMed/MEDLINE.

**Results:**

Romantic rejection and loss activate brain regions involved in emotion regulation and pain (vlPFC, AI, ACC), overlapping with patterns seen in depression and addiction. Women tend to show stronger limbic and stress responses, while men engage more prefrontal and reward circuits, reflecting different coping styles. People with insecure attachment or high rejection sensitivity experience more intense emotional and physiological distress. Clinically, romantic loss is linked to depression, anxiety, rumination, and sometimes borderline personality features.

**Conclusions:**

Romantic suffering involves brain and psychological processes linked to emotional disorders. Gender influences these neural and behavioral responses, shaping vulnerability and coping. Recognizing these patterns can inform gender-sensitive prevention and treatment. Future research should use longitudinal and experimental methods to clarify how romantic distress leads to psychopathology.

**Disclosure of Interest:**

None Declared

